# Effect of multilayer high-compression bandaging on ankle range of motion and oxygen cost of walking

**DOI:** 10.1258/phleb.2011.010084

**Published:** 2012-02

**Authors:** K S Roaldsen, B Elfving, J K Stanghelle, E Mattsson

**Affiliations:** * Karolinska Institutet, Department of Neurobiology, Care Sciences and Society, Division of Physiotherapy, Stockholm, Sweden; † Sunnaas Rehabilitation Hospital and Faculty of Medicine, University of Oslo, Oslo, Norway

**Keywords:** ankle mobility, compression, gait efficiency, oxygen consumption, own opinions

## Abstract

**Objective:**

To evaluate the effects of multilayer high-compression bandaging on ankle range of motion, oxygen consumption and subjective walking ability in healthy subjects.

**Method:**

A volunteer sample of 22 healthy subjects (10 women and 12 men; aged 67 [63–83] years) were studied. The intervention included treadmill-walking at self-selected speed with and without multilayer high-compression bandaging (Proforeº), randomly selected. The primary outcome variables were ankle range of motion, oxygen consumption and subjective walking ability.

**Results:**

Total ankle range of motion decreased 4% with compression. No change in oxygen cost of walking was observed. Less than half the subjects reported that walking-shoe comfort or walking distance was negatively affected.

**Conclusion:**

Ankle range of motion decreased with compression but could probably be counteracted with a regular exercise programme. There were no indications that walking with compression was more exhausting than walking without. Appropriate walking shoes could seem important to secure gait efficiency when using compression garments.

## Introduction

Compression treatment has been used for centuries to manage varicose veins, the post-thrombotic syndrome, leg ulcers and also, in modern times, for preventing deep vein thrombosis. The mechanisms of compression treatment are not clear, but the beneficial effects are increased lymphatic flow and venous return, improved cutaneous microcirculation, restored valvular competence and improved effect of the calf muscle pump.^1^ Recent improvement in compression bandages involve the use of multilayer bandages with high pressure to be exerted and sustained to the lower leg, with the possibility of narrowing the margin between the benefit of compression therapy and the possibility of hampering patients' mobility/activity. Multilayer compression systems are found to be most effective in ulcer healing and are widely used.^3^ Effects on ankle joint mobility or gait capacity have to our knowledge not been studied before.

In patients with leg ulcer, lifelong compression treatment is crucial to increase venous return, heal ulcers and prevent ulcer recurrence.^2,3^ In addition to compression, the initial treatment of patients with leg ulcer should be exercise combined with leg elevation, and use of adequate clothing and shoes.^4,1^ A general low level of physical activity, reduced walking speed, impaired ankle range of motion and associated poor calf pump function are reported in these patients in comparison with age-matched controls.^5–7^ This might, among other reasons, indicate problems with regard to being physically active.

The major practical difficulty of compression treatment, and also the best predictive factor for ulcer resistance or recurrence, is reportedly non-compliance of treatment by the care-giver or the patient.^8^ Patients with leg ulcer have reported restricted ankle range of motion and distorted walking ability because of ‘bulky and strenuous’ compression garments.^9^ This indicates that compression bandaging might ultimately restrict ankle range of motion and gait capacity, thereby reducing calf-pump effectiveness.

The aim of this study was to evaluate the effects of multilayer high-compression bandaging on ankle range of motion and oxygen cost of walking in subjects without musculoskeletal problems, and also to note subjective opinions of compression on walking ability and walking-shoe comfort.

## Methods

### Subjects

Subjects without muscular-skeleton problems in the lower limbs were recruited through colleagues, friends and neighbours. Mild venous insufficiency (varicose veins) was not an exclusion criterion as long as walking ability was unaffected. Information about the study was given personally or by mail or telephone. Prior to entry, the test subjects received written information and gave their informed consent. Ethical approval was obtained from the Regional Ethical Review Board in Stockholm.

A convenient sample of 22 volunteer subjects were included, 10 women and 12 men. The median age was 67 (63–83) years. Demographic and background variables are shown in Table 1. Six of the subjects had varicose veins among whom four used class II compression stockings regularly (two during winter only, two all year long).

**Table 1:** Demographic and background variables of the subjects (*n* = 22)

Gender	*N*	Age (years)	BMI (kg·m^−2^)
Female	10	67 (63–70)	21.6 (18.8–25.6)
Male	12	68 (64–83)	24.6 (21.9–26.7)
Total	22	67 (63–83)	23.6 (18.8–26.7)

#### Compression

**Figure 1:**
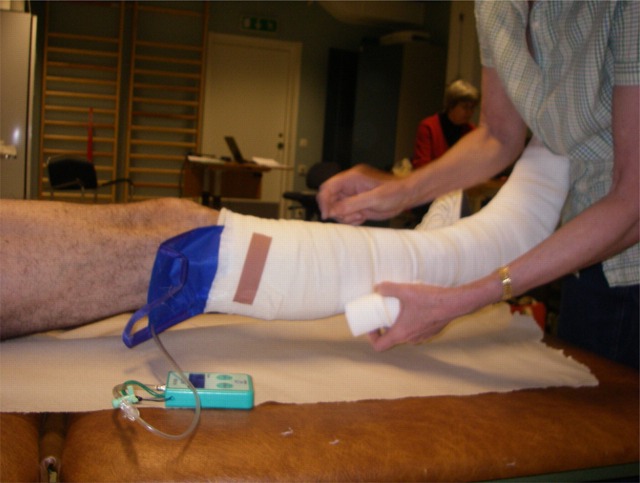
Measuring sub-bandage pressure using the Kikuhime pressure transducer (Biomedical Systems Engineering). The pressure transducer probe is placed inside a blue sheet positioned under the bandage to make it possible to slide the probe underneath the bandage from the ankle to the knee measuring sub-bandage pressure at three points: the lateral malleolus, 1.5 cm below caput fibulae and at the knee

A multilayer high-compression system (Proforeº, Smith & Nephew) was chosen. The bandage was applied by an experienced nurse according to the supplier's instructions (see http://wound.smith-nephew.com/se/Product.asp?NodeId=857&Tab=5&Hide=,14August2008). To ensure that the level of compression was equal in all subjects with a falling gradient from the ankle to the knee, a sub-bandage pressure transducer (Kikuhime, Biomedical Systems Engineering, large probe) was used (Figure 1). The transducer probe was placed inside a sheet positioned under the bandage, which made it possible to slide the probe underneath the bandage from the ankle to the knee. The sub-bandage pressure was measured at three different points; at the lateral malleolus, 15 cm below caput fibulae, and at the knee. The measurements were made in a prone position prior to treadmill-walking. Median sub-bandage pressures in the subjects were 40 (38–42) mmHg, 30 (28–32) mmHg and 20 (19–21) mmHg, respectively.

### Measurements

All measurements were performed by an experienced team – a physiotherapist, a bio-engineer and a nurse – at the Movement Laboratory at Karolinska University Hospital, Solna, Stockholm, Sweden, on two different days with a median of six (0.5–57) days apart. The subjects were instructed to bring exercise clothing and the same preferred walking shoes on both occasions. Demographic and background variables were reported by the subjects.

The International Classification of Functioning, Disability and Health (ICF) was used as a conceptual model.^10^ The following components of ICF were included: ‘body functions and structures’, ‘activities’ and ‘personal factors’.

### Body functions and structures

#### Ankle range of motion (primary outcome)

Loaded dorsiflexion of the ankle was measured in a standing position with one leg on the floor and the examined leg on a 30-cm-high box.^11^ The subject leaned forward with the sole of the examined foot flat on the box and the greater part of the body weight on this foot to the point where the heel was just in contact with the surface of the box. The angle between the box surface and a line through the tip of the lateral malleoleus and the head of the fibula was assessed using a standard goniometer.

To measure plantar flexion the subject sat on the edge of a chair. The investigated leg was stretched forward with the sole of the foot on the floor to the point where the medial part of the forefoot was just in contact with the floor.^12^ The angle between the floor and the lateral-malleolus and head-of-fibula line was measured with a standard goniometer.

The normal value for loaded dorsiflexion (with bent knee) is stated as 30° and for plantar flexion (with straight knee) as 37.6°, 36 in men and 39 in women.^13,11^ Ankle range of motion was measured both with and without compression. All measurements were repeated twice to ensure accuracy and the best value was chosen.

#### Oxygen cost (primary outcome)

**Figure 2:**
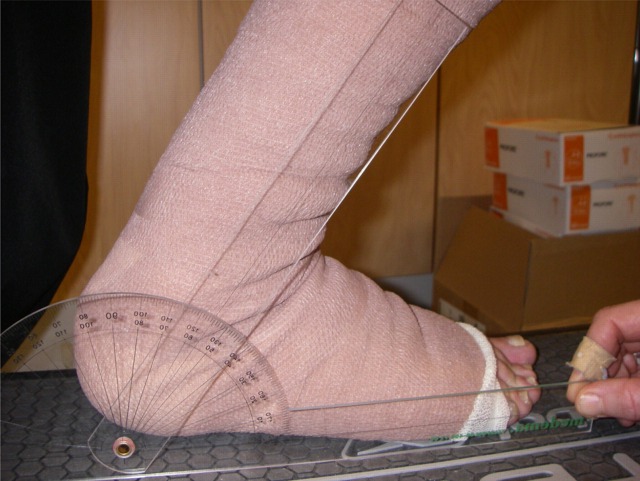
Measuring loaded ankle dorsiflexion with a Goniometer

**Figure 3:**
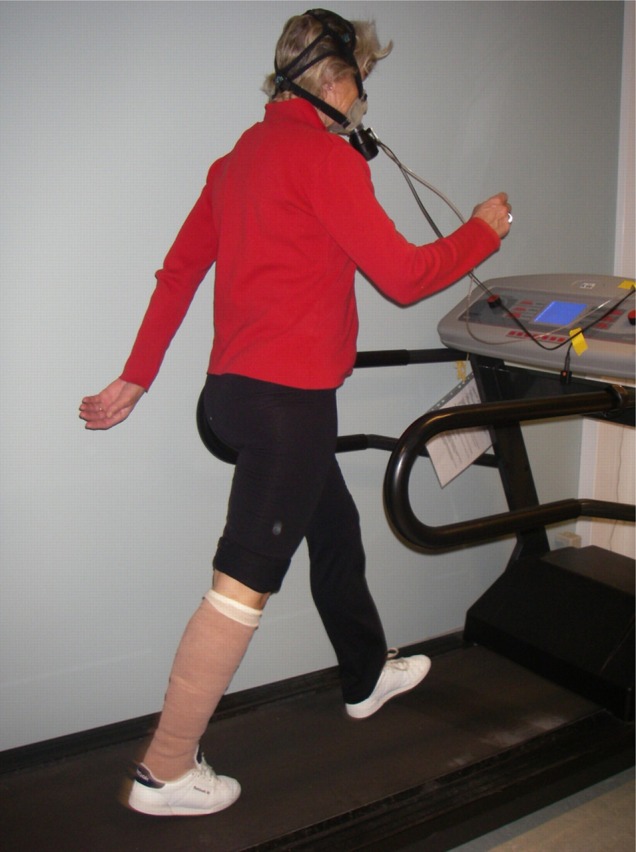
Measuring oxygen consumption while walking on a treadmill with multilayer high-compression bandaging on one leg

Oxygen consumption, VO_2_ (l minute^−1^) was assessed after six minutes, at steady state, while the subject was walking on a treadmill (Cardio Rehab-Hill 3175, Cardionics Försäljning AB) with and without compression bandaging at a speed as close as possible to the self-selected walking speed in the corridor (Figure 2). Metamax II (Serial nos. M II 53229901) was used for assessing oxygen consumption. In Metamax II, O_2_ and CO_2_ are measured in the expired air with a turbine flow meter attached to a breathing mask. Sampled expired air is equilibrated with surrounding air before the fractions of O_2_ and CO_2_ are measured. The instrument was used according to the instructions in the manual and was calibrated against a commercial gas of known concentration of O_2_ and CO_2_ before the start of the experiment. The instrument was further calibrated against room air, the concentration of O_2_ and CO_2_ in the room air being read and the flow transducer calibrated with a 3 l high-precision calibration syringe (Calibration syringe D, Sensomedics) before each trial with a new subject. During the experiments the data collected were immediately transferred to and stored in a computer calculating the oxygen cost in mL · kg^−1^ · minute^−1^ and in mL · kg^−1^ · m^−1^. Heart rate was monitored using a heart rate monitor with a chest transmitter (Polar, Polar Oy, Kempele, Finland) (Figure 3).

#### Estimated maximal oxygen capacity

Maximal oxygen capacity (VO_2_ max) was estimated by means of a sub-maximal bicycle-ergometry test according to Åstrand and Rhyming.^14^ The work rate on the bicycle (Monark Ergomedic 839) was adjusted depending on the subject's gender, age and physical activity level to reach the required heart rate that is above 120 beats/minute. The subjects cycled at the final work load for at least six minutes to reach steady state. Heart rate was monitored using a heart rate monitor with a chest transmitter (Polar, Polar Oy, Kempele, Finland). Maximal oxygen capacity (VO_2_max, l · minute^−1^) was calculated according to the nomogram and the factors provided by Åstrand and Rhyming and Åstrand and Rodahl.^14,15^ Relative VO_2_max (mL · kg^−1^ · minute^−1^) was calculated from absolute VO_2_max ( l · minute^−1^) and measured body weight (kg).

### Activities

#### Self-selected walking speed

Self-selected walking speed over a level floor was assessed using a modified 6-minute walking test (6MWT).^16,17^ The subjects walked along a 35 m calm indoor corridor with marks every meter. They were instructed to walk for six minutes without running or jogging, using their customary walking speed. Information regarding how many minutes they had walked and minutes to go were given each minute during the test.^18^ Exertion was rated on the Borg RPE scale (6–20) after the test, the total walking distance was noted and the walking speed was calculated.^19^

### Personal factors

#### Subjects' opinions (primary outcome)

The following questions were asked when the subjects had walked with and without compression: ‘Suppose you were to walk for a longer distance. Would you be able to walk the same distance with and without compression? Does the compression garment affect walking comfort in your regular walking shoes?’

#### Test occasions 1 and 2

During the first test occasion the subjects performed the sub-maximal bicycle-ergometry test and the 6MWT. After the tests the subjects were instructed in treadmill-walking and the breathing mask was introduced as the participants were not familiar with treadmill walking. Walking speed was successively increased as close as possible to the speed chosen in the corridor. Plantar flexion and dorsiflexion were explained and measured.

During the second test occasion, oxygen consumption was assessed while the subjects walked thrice on the treadmill, first without and then with or without compression, randomly chosen. The subjects rested in a prone position for 10 minutes after each session. Ankle range of motion was measured prior to walking on the treadmill both with and without compression. Questions regarding the subjective experience of compression on walking ability and walking-shoe comfort were asked after the last walking session.

### Statistics

Ratio data are presented as median and range, and nominal data by numbers/frequencies. The rank test was used to test differences between walking with and without compression.

A *P* value of ≤0.05 was considered statistically significant. All statistical analyses were performed with the SPSS version 15.0 (SPSS Inc, Chicago, IL, USA).

## Results

### Body functions and structures

#### Ankle range of motion (primary outcome)

The median value for total ankle range of motion (the sum of plantar flexion and dorsiflexion) without compression was 87 (69–108)°, decreasing to 83 (68–105)° (*P* = 0.001) with compression bandaging. The median decrease in the total ankle range of motion was 3.3 (−12–4)° (4%). The reduction in the total ankle range of motion was primarily due to reduced ankle-dorsiflexion (Table 2).

**Table 2:** Results from ankle range of motion, floor-walking, treadmill-walking and bicycle-ergometry tests (*n* = 22)

	Without compression		With compression		
	Median	Range	Median	Range	*P* value
*Primary outcome variables*					
**Ankle range of motion**					
Total ankle range of motion (°)	87	69–108	83	68–105	0.001
Dorsiflexion (°)	36	24–49	34	22–47	0.004
Plantar flexion (°)	51	39–61	50	34–60	0.175
**Treadmill-walking**					
Self-selected walking speed (m·s^−1^)	1.32	1.11–1.50	1.32	1.11–1.50	
Oxygen consumption, VO_2_ (l·minute^−1^)	0.86	0.49–1.94	0.84	0.54–1.49	0.974
Oxygen cost, VO_2_/kg					
mL·kg^−1^·minute^−1^	11.9	9.4–17.0	12.2	9.7–18.2	0.465
mL·kg^−1^·m^−1^	0.156	0.13–0.23	0.152	0.13–0.24	0.505
Heart rate (beats·minute^−1^)	87	70–113	86	71–117	0.182
Perceived exertion (6–20)	11	9–13	11	9–14	0.491
*Descriptive variables*					
**Floor-walking**					
Self-selected walking speed (m·s^−1^)	1.47	1.17–1.69			
Perceived exertion (6–20)	11	9–13			
**Bicycle-ergometry test**					
Work load (kpm · minute^−1^)	450	375–900			
Heart rate (beats · minute^−1^)	128	102–147			
Maximum oxygen capacity, VO_2_max					
l · minute^−1^	1.7	1.3–3.1			
mL · kg^−1^ · minute^−1^	28.8*	19.0–45.5			
Perceived exertion (6–20)	15	12–17			

*Ergometry reference values for subjects 60–70 years are 27–36 mL · kg^−1^ · minute^−115^

#### Oxygen cost (primary outcome)

**Figure 4:**
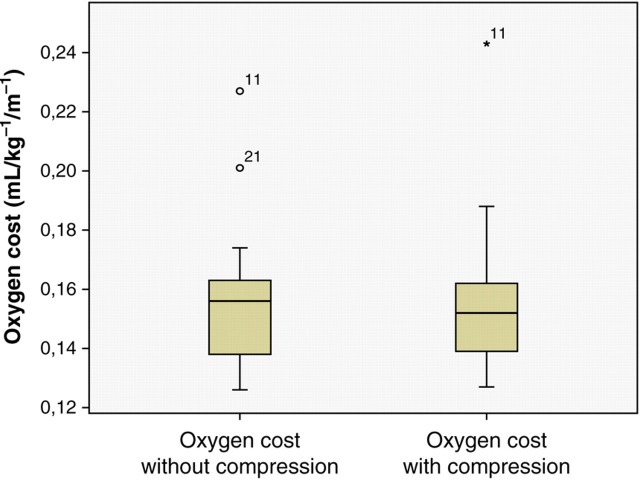
Box plot, first and third quartile and range of oxygen cost (mL · kg^−1^ · m^−1^) in subjects (*n* = 22) walking with and without four-layered high-compression bandaging (*P* = 0.505)

The median values for oxygen cost of walking without and with compression were 0.156 (0.13–0.23) mL · kg^−1^ · m^−1^ and 0.152 (0.13–0.24) mL · kg^−1^ · m^−1^ (*P* = 0.505), respectively. Oxygen costs of walking with and without compression for the 22 test subjects are shown in Figure 4.

The relative oxygen costs of treadmill-walking with and without compression, % VO_2_ max of walking [=(VO_2_ walking/VO_2_ bicycling) × 100] were calculated to 43 (29–64)% without and 43 (27–72)% with compression (*P* = 0.506).^15^

#### Estimated maximal oxygen capacity

The median value of calculated maximal oxygen capacity, VO_2_ max · kg^−1^, 28.8 (19.0–45.5) mL · kg^−1^ · minute^−1^, was equal to age- and gender-corrected reference values (Table 2).^15^

### Activities

#### Self-selected walking speed

Self-selected walking speed along the corridor was 1.47 (1.17–1.69) m · s^−1^ (Table 2). Customary walking speed in seniors 60–70 years is reported to be 1.23 (1.0–1.67) m · s^−1^.^20^ Self-selected walking speed on the treadmill, 1.32 (1.11–1.50) m · s^−1^, was significantly lower than self-selected floor-walking speed (*P* = 0.0001). There were no differences in perceived exertion between floor- and treadmill-walking (Table 2).

### Personal factors

#### Subjects' opinions (primary outcome)

Nine subjects experienced problems fitting the bandaged foot into their regular walking shoes and stated that shoe comfort was affected, while five reported that walking ability was reduced in such a way that they were unable to walk the same distance with compression as without.

## Discussion

The main findings in this study were that the total ankle range of motion showed a 4% decrease with compression, and that dorsiflexion was more affected than plantar flexion but that walking with or without compression showed no difference in oxygen cost. Furthermore, compression bandaging negatively affected walking comfort or walking ability in less than half of the subjects.

That total ankle range of motion was decreased with compression is in accordance with Lentner *et al.* ^21^ who found that compression bandaging restricted ankle joint mobility between 7% and 32% in healthy subjects, and that thick bandages led to a more pronounced decrease than thin ones. The reduction in our study was more modest. However, the results cannot be compared as the measurement methods differed in the two studies. Lentner *et al.* used a passive measurement without loading on the foot, while we used a loaded method that enabled the subjects to use their bodyweight to stretch the bandages, thus giving better mobility measurements. Ankle mobility in loaded dorsiflexion is considered to be the clinical test that correlates best with end results after ankle problems such as immobilization and fractures.^12^ Compression systems have developed during the past 12 years, which may also explain our better results.

A 4% reduction in ankle mobility is probably of minor clinical significance in healthy subjects with normal ankle range of motion, but might be clinically significant for leg-ulcer patients, especially dorsiflexion-reduction. The total ankle range of motion is about 58–68° (depending on the method of measurement), which in patients with active ulceration is curtailed to an average of only 21°.^5^ Normal gait on level ground requires approximately 10° of dorsiflexion during the stand phase and toe-off, and approximately 20–30° are needed during stair-walking, knee flexion or exercise.^22^

Even a small additional reduction in ankle range of motion due to a compression bandage, can mean the difference between being able or not able to climb stairs or walk on uneven ground, thus impairing the calf muscle pump function and ulcer healing. This has been demonstrated by Barwell *et al.* ^23^ In their study, only 13% of patients who had less than 35° of ankle range of motion had healed at 24 weeks, compared with 60% of those with more than 35°. Thus, partial immobilization should be counteracted with a regular exercise programme. Davies *et al.* ^24^ showed that a simple, home-based exercise programme was effective in improving ankle-range of motion in patients with long-term venous ulcer. Compression stockings, which cause little restriction of mobility, should preferably be used when suitable.^25^

We found no difference in oxygen cost between walking with and without compression bandaging at speeds between 1.1 and 1.5 m · s^−1^. To our knowledge there are no published studies of energy expenditure in walking with lower-leg compression. In patients with orthopaedic diseases Mattsson and Broström^26^ found that energy cost of walking was increased by 10% with an unstable ankle compared with normal conditions for healthy subjects. Waters *et al.* ^27^ found that individuals with ankle fusion required 3% higher energy expenditure than normal subjects walking at the same speed. The present compression system has probably no effects on the energy expenditure during walking in healthy subjects. Experimental data from a sample of six patients with venous leg ulcer indicate that this is also the case in patients.^28^

Oxygen cost of walking is obviously a parameter which is not sensitive enough to show any clinical implication of reduced ankle mobility. There is a need for concentrating on outcome measures like venous pumping function when studying the effect of restricted ankle mobility due to compression in patients with venous leg ulcer.^29^ Kügler *et al.* ^30^ found that artificially restricting the movement of the ankle joint decreased the effectiveness of the calf muscle pump during walking in healthy subjects. This is probably also the case in patients with venous leg ulcer.

The relative oxygen costs during walking with and without compression were calculated to 43% of VO_2_ max both with and without compression. This finding tallies with that of Waters and Mulroy^20^ who stated that the rate of oxygen consumption at self-selected walking speed requires nearly 48% of the VO_2_ max in a subject 75 years of age. Walking requires little effort as long as it taxes less than 50% of VO_2_ max.^20^ Patients with leg ulcer are accustomed to wearing compression bandaging, which was not the case for our test subjects, indicating that walking with compression might even be less affected in patients.

In this study, maximal oxygen capacity during walking was calculated from the predicted VO_2_ max from a sub-maximal bicycle-ergometry test. A maximal test or a sub-maximal walking test on a treadmill had been preferable. This was not done, both for convenience and for exposing the subjects to a minimum of effort.

Treadmill-walking speed was significantly lower than floor-walking speed, yet not different from adults' customary walking speed, which is between 1.0–1.67 m · s^−1^.^20^ The lowered treadmill-walking speed in our study was probably a result of the subjects' high age and their feeling of insecurity, as they had no experience of treadmill-walking prior to the two days of testing. However, the learning effect probably did not affect the test results, as the test order was randomized.

We chose to measure energy expenditure at a controlled walking speed on a treadmill to guarantee the same speed both with and without compression. Self-selected walking speed differs among patient populations depending on the extent of disability.^20^ Patients with gait disabilities, often seen in patients with leg ulcer, have difficulties adjusting to walking on a treadmill. It might be preferable to test patients on a track, allowing them to select their preferred walking speed and use their customary walking aids. However, the walking speed would be difficult to standardize under those conditions.

Distorted walking ability because of compression garments has previously been reported in patients with leg ulcer.^9^ In the present study, reduced walking-shoe comfort, problems using regular walking shoes and impaired walking ability were reported in nearly half the subjects. To facilitate recommended walking in patients with venous leg ulcer, the use of appropriate walking shoes in combination with compression garments should be stressed.

## Conclusion

Ankle range of motion was decreased with compression bandaging. There were no differences in oxygen cost of walking with and without compression. Compression bandaging negatively affected walking comfort due to problems with walking shoes in nine subjects and five reported that walking ability was impaired due to the compression garment.

## Implications

Compression bandages reduce ankle range of motion and should be counteracted with a regular exercise programme. Compression stockings, which cause little restriction of ankle range of motion, should preferably be used when suitable. The results indicate that walking with compression is not too exhausting and can be recommended as a part of the treatment strategy for patients with leg ulcer. The use of appropriate walking shoes in combination with compression garments is important to secure gait efficiency and comfortable walking.

## References

[1] Alguire PC, Mathes B. Chronic venous insufficiency and venous ulceration. J Gen Intern Med 1997;12:374–83 9192256 10.1046/j.1525-1497.1997.00063.x PMC1497122

[2] Fletcher A, Cullum N, Sheldon T. A systematic review of compression treatment for venous leg ulcers. BMJ 1997;315:576–80 9302954 10.1136/bmj.315.7108.576 PMC2127398

[3] Cullum N, Nelson EA, Fletcher AW, Sheldon TA. Compression for venous leg ulcer. Cochrane Database Syst Rev 2001;2:CD000265. Updated in *Cochrane Database Syst Rev* 2009;**1**:CD000265 10.1002/14651858.CD000265 11405957

[4] Franks PJ, Mofatt CJ, Connolly M, Factors associated with healing leg ulceration with high compression. Age Ageing 1995;24:407–10 8669344 10.1093/ageing/24.5.407

[5] Back TL, Padberg FT, Araki CT, Thompson PT, Hobson RW. Limited range of motion is a significant factor in venous ulceration. J Vasc Surg 1995;22:519–23 7494349 10.1016/s0741-5214(95)70030-7

[6] Roaldsen KS, Rollman O, Torebjörk E, Stanghelle JK, Olsson E. Functional ability in female patients with leg ulcers – a challenge to physiotherapy. Physiother Res Int 2006;11:191–203 17236527 10.1002/pri.337

[7] Heinen MH, van der Vleuten C, de Rooij MJM, Uden CJT, Rvers AWM, van Achterberg T. Physical activity and adherence to compression therapy in patients with venous leg ulcer. Arch Dermatol 2007;143:1283–8 17938342 10.1001/archderm.143.10.1283

[8] Raju S, Hollis K, Neglen P. Use of compression stockings in chronic venous disease: patient compliance and efficacy. Ann Vasc Surg 2007;21:790–5 17980798 10.1016/j.avsg.2007.07.014

[9] Persoon A, Heinen MM, van der Vleuten CJ, de Rooij MJ, van de Kerkhof PC, van Achterberg T. Leg ulcers: a review of their impact on daily life. J Clin Nurs 2004;13:341–54 15009337 10.1046/j.1365-2702.2003.00859.x

[10] WHO, World Health Organization. International Classification of Functioning. Geneva: Disability and Health (ICF), 2001.

[11] Lindsjö U, Danckwardt-Lilliestrom G, Sahlstedt B. Measurement of the motion range in the loaded ankle. Clin Orthop Rel Res 1985;199:68–71 4042498

[12] Nilson G, Nyberg P, Ekdahl C, Eneroth M. Performance after surgical treatment of patients with ankle fractures – 14-month follow-up. Physiother Res Int 2003;8:69–82 12879729 10.1002/pri.274

[13] Livingstone L, Stevenson J, Olney S. Stair climbing kinematics in stairs of different dimensions. Arch Phys Med Rehabil 1991;72:398–402 2059107

[14] Åstrand PO, Rhyming I. A nomogram for calculation of aerobic capacity (physical fitness) from pulse rate during sub-maximal work. J Appl Physiol 1954;7:218–21 13211501 10.1152/jappl.1954.7.2.218

[15] Åstrand PO, Rodahl K. Textbook of Work Physiology. Physiological Bases of Exercise. 2nd edn. New York: McGraw Hill, 1977

[16] Butland RJA, Pang J, Gross ER, Woodcock AA, Geddes DM. Two, six, and 12-minute walking tests in respiratory disease. BMJ 1982;284:1604–8 6805625 10.1136/bmj.284.6329.1607 PMC1498516

[17] Guyatt GH, Sullivan MJ, Thompson PJ, The six-minute walk. A new measure of exercise capacity in patients with chronic heart failure. Can Med Assoc J 1985;132:919–23 3978515 PMC1345899

[18] American Thoracic Society (ATS). ATS statement: guidelines for the six-minute walk test. Am J Respir Crit Care Med 2002;166:111–7 12091180 10.1164/ajrccm.166.1.at1102

[19] Borg GAV. Psychophysical bases of perceived exertion. Med Sci Sports Exerc 1982;14:377–81 7154893

[20] Waters RL, Mulroy S. The energy expenditure of normal and pathological gait. Gait Posture 1999;9:207–31 10575082 10.1016/s0966-6362(99)00009-0

[21] Lentner A, Späth F, Wienert V. Limitation of movement in the ankle and talo-calcaneonavicular joints caused by compression bandages. Phlebology 1997;12:25–30

[22] Inman VT, Ralston HJ, Todd F. Human Walking. Baltimore, MD: Williams and Wilkins, 1981

[23] Barwell JR, Taylor M, Deacon J, Davis C, Whyman MR, Poskitt KR. Ankle mobility is a risk factor for healing of chronic venous leg ulcer. Phlebology 2001;16:38–40

[24] Davies JA, Bull RH, Farrelly IJ, Wakelin MJ. A home-based exercise programme improves ankle range of motion in long-term venous ulcer patients. Phlebology 2007;22:86–9 18268857 10.1258/026835507780346178

[25] Lentner A, Späth F, Wienert V. Influence of compression hosiery on the mobility of the ankle joint and the talo-calcaneonavicular joint. VASA J Vasc Dis 1996;25:60–4 8851267

[26] Mattsson E, Broström LÅ. The increase in energy cost of walking with an immobilized knee or an unstable ankle. Scand J Rehabil Med 1990;22:51–3 2326610

[27] Waters RL, Barnes G, Husserel T, Silver L, Liss R. Energy expenditure following hip and ankle arthrodesis. J Bone Joint Surg 1988;70A:1032–7 3403571

[28] Roaldsen KS. *Factors influencing physical activity in patients with venous leg ulcer*. Doctoral thesis, Karolinska Institutet, Stockholm, 2009

[29] Nelzén O. Prevalence in venous leg ulcer: the importance of the data collecting method. Phlebolymphology 2008;15:143–50

[30] Kügler C, Strunk M, Rudofsky G. Venous pressure dynamics of the healthy human leg: role of muscle activity, joint mobility and anthropometric factors. J Vasc Res 2001;38:20–9 11173991 10.1159/000051026

